# Enhancing UV-C and perchlorate resistance in *Arabidopsis thaliana* through the introduction of microbial genes from hypersaline environment

**DOI:** 10.3389/fmicb.2026.1789302

**Published:** 2026-04-15

**Authors:** Carolina González de Figueras, Sara Gómez, Maria Lamprecht-Grandío, Salvador Mirete, Jorge Díaz-Rullo, Pablo Martínez-Rodríguez, Mercedes Sánchez-Costa, José Eduardo González-Pastor

**Affiliations:** 1 Department of Molecular Evolution, Astrobiology Center (CAB), (CSIC-INTA), Madrid, Spain; 2 Atlántico Medio University, Las Palmas de Gran Canaria, Spain

**Keywords:** abiotic stress tolerance, climate change adaptation, extremophiles, microbial genes, oxidative stress, plant stress response, sodium perchlorate, UV-B/UV-C radiation

## Abstract

Ultraviolet (UV) radiation reaching the Earth's surface affects all living organisms. Recent reports show a trend of increasing exposure levels due to stratospheric ozone depletion and contamination. UV-B radiation (280–315 nm), previously largely absorbed by the ozone layer, now reaches the surface in higher doses, posing a particular threat to plants, which are sessile organisms and cannot escape adverse conditions. The intrinsic protective and repair mechanisms in plants may be insufficient to counteract this increase, potentially impacting crop productivity, distribution, and quality, with serious implications for agriculture and ecological stability. This study aims to enhance plant resistance to UV radiation by introducing genes derived from extremophilic microorganism, which have previously shown to confer UV-protective effects in UV resistance to a radiation-sensitive *Escherichia coli* strain (*recA* mutant). Extremophile microorganisms have been discovered in high-irradiation environments, such as hypersaline lakes, where survival relies on unique genetic adaptations. In our laboratory, four genes were selected from metagenomic libraries derived from high-altitude hypersaline lakes in Argentina (Diamante and Ojo Seco, at 4,589 m and 3,200 m respectively) and from the Es Trenc salt flat (Mallorca, Spain). Based on these promising results, the genes were introduced into *Arabidopsis thaliana* to evaluate their potential to enhance UV-B tolerance in plants. The selected genes included one encoding a TATA-box binding protein, and three hypothetical proteins. Each gene was independently transformed into *Arabidopsis thaliana* lines and subjected to UV-B and UV-C irradiation (4.5 kJ·m^−2^), with UV-C (100–280 nm) ultimately chosen for its higher damaging potential to test the limits of plant tolerance. Additionally, cross-resistance was evaluated using sodium perchlorate, a common soil contaminant and oxidative stressor. Plants were exposed to concentrations between 3.67 and 7.34 g/L, exceeding those used in previous studies. As a result, the plants obtained were more resistant to UV radiation and were also capable of growing in environments containing higher levels of perchlorate in the growth medium. Thus, the expression of these genes in the plant appears to contribute to enhanced stress resistance.

## 1 Introduction

Living organisms on Earth are continuously exposed to solar radiation, which includes ultraviolet (UV) light. Ultraviolet radiation, a component of the non-ionizing portion of the electromagnetic spectrum, representing approximately 7% of the total solar radiation reaching the Earth's surface (Mintoff et al., 2015). It is considered one of the most harmful environmental factors for cellular components and is classified among major abiotic stressors alongside extreme temperatures, high salinity, and heavy metal exposure (Ibrahimova et al., 2021; Kumari et al., 2020). According to the International Commission on Illumination (1987) and Sliney (2007), UV radiation is categorized into three wavelength ranges: UV-A (315-400 nm), UV-B (280-315 nm), and UV-C (100–280 nm).

UV-A, which constitutes the largest proportion of UV radiation reaching the Earth's surface (~6.8%), is generally considered the least biologically harmful. In contrast, UV-B, although comprising only ~2.8% of the UV spectrum, can induce a broad range of detrimental effects in plant systems. However, these effects may be attenuated when UV-B exposure occurs together with longer wavelengths, such as UV-A and/or visible light (Bornman et al., 2019), and are further reduced by absorption in the stratospheric ozone layer (Bernhard et al., 2020; Dawood et al., 2022). UV-C radiation, the most energetic and potentially most damaging band, is almost completely absorbed by stratospheric ozone (O_3_) and atmospheric oxygen (O_2_). As a result, UV-C at ground level is considered negligible (~0.8%) (Douki, 2020; Madronich et al., 1998).

Although the ozone layer has shown signs of recovery in recent decades, UV-B radiation levels have not decreased proportionally (Bais et al., 2015) This discrepancy arises from the combined influence of several factors beyond stratospheric ozone concentration, including cloud cover, surface albedo, aerosols, solar activity, solar emissions, and the geometric configuration of the Earth-Sun system (Fröhlich and Lean, 2004; McKenzie et al., 2007; Shaffer and Cerveny, 2004). Moreover, recent evidence suggests that UV-B levels are increasing in some regions (Liaqat et al., 2024), with direct implications for plant biology and agricultural productivity. Elevated UV-B exposure influences species distribution along altitudinal and latitudinal gradients, exposing plants to novel combinations of abiotic and biotic stressors that may compromise their productivity (WMO, Scientific Assessment of Ozone Depletion, 2018) Additionally, UV-B can impair plant resistance to pests and diseases, alter nutritional quality, and decrease crop yield (Bornman et al., 2019). UV-B induced morphological changes may also modify competitive interactions between crops and weeds, with non-native species often exhibiting greater acclimation capacity to environmental changes than native flora (Alexandru Suchar and Robberecht, 2018; Barnes et al., 2005; Davidson et al., 2011).

Beyond their ecological consequences, these shifts pose a significant threat to global food security. With the global population projected to reach 9 billion by 2050 (Novotny et al., 2023), maintaining stable and sufficient food production has become a critical global priority. Increasing evidence shows that elevated UV-B radiation negatively affects major cereal crops-fundamental staples of the human diet (Nayik et al., 2023). Wheat and maize, in particular, exhibit marked reductions in quality and yield (Li et al., 2010; Wijewardana et al., 2016), while rice shows declines in total biomass and productivity (Mohammed et al., 2007). Perchlorate, a widespread environmental contaminant present in water, soil, and food, also reduces plant growth parameters due to its strong oxidative and metabolic disruptive effects. This potent oxidant is derived primarily from activities associated with the defense sector, as well as industrial and aerospace processes (Backus et al., 2005; He et al., 2013) Nevertheless, a fraction can be attributed to natural formation under specific geochemical conditions. It is also present as a component of certain naturally occurring fertilizers, such as Chilean nitrate deposits (Seyfferth and Parker, 2008; Vega et al., 2018). Therefore, resistance to this compound and/or its effective removal from the environment and agriculture is critical.

Chemically, this inorganic anion–characterized by a delocalized negative charge-, consists of a chlorine atom in oxidation state surrounded by four oxygen atoms (Urbansky, 1998). It is highly soluble in water, chemically stable, and environmentally persistent (He et al., 2013). Due to its low absorption into the soil, it is readily transported through aquatic systems (Coates and Achenbach, 2004). This compound promotes macromolecular denaturation, induces oxidative stress, and causes DNA damage across a wide range of terrestrial organisms, while in humans it additionally disrupts thyroid gland function, potentially leading to hypothyroidism or iodine deficiency (Kumarathilaka et al., 2016; Zhang and Cremer, 2010).

UV radiation can directly damage DNA by inducing photo damage such as cyclobutane pyrimidine dimers (CPDs) and (6-4) photoproducts [(6-4)PPs]. Indirectly, it stimulates the production of reactive oxygen species (ROS) and reactive nitrogen species (RNS), which in turn generate oxidative DNA damage, including the formation of 8-oxo-7,8-dihydro2′-deoxyguanosine (8-oxo-dG) and 2,6-diamino-4-hydroxy-5-formamido-pyrimidine (Fapy) (Banaś et al., 2020; Gill et al., 2015; Kumari et al., 2022). UV radiation also affects protein integrity by inactivating enzymes and structural proteins (Grossweiner, 1984; Prinsze et al., 1990). In oxygen-rich environments, it promotes lipid peroxidation, compromising the integrity of unsaturated fatty acids in plant cell membranes (Kramer et al., 1991; Kumari et al., 2022; Panagopoulos et al., 1990).

Due to their sessile nature, plants are particularly vulnerable to ultraviolet (UV) radiation. While they rely on broad range of electromagnetic wavelengths(280-100,000 nm) for essential biological processes, plants also use specific portions of the spectrum as sources of information through photoreceptors that regulate metabolism, development, and viability throughout the life cycle (Bornman et al., 2019; Jansen et al., 1998; Jenkins, 2009, 2017). Upon exposure to UV-B radiation, plants activate a suite of defense responses that include the upregulation of photo repair mechanisms, enhanced of antioxidant capacity through enzymatic systems–such as superoxide dismutase (SOD), glutathione (GSH), catalase (CAT), redoxins and non-enzymatic antioxidants including ascorbate, tocopherol, glutathione, and phenolic compounds (Dias et al., 2020). Plants also induce the accumulation of UV-screening mechanisms such asflavonoids and carotenoids, which attenuate UV penetration into sensitive tissues (Liaqat et al., 2024). The evolutionary refinement of these protective mechanisms enables plants to maintain a dynamic equilibrium between damage and repair, ultimately shaping their overall sensitivity and adaptative capacity to UV stress (Banaś et al., 2020).

Traditionally, research has focused primarily on the biological effects and underlying mechanisms of UV-A and UV-B radiation, while the impacts of UV-C have received comparatively less attention (Urban et al., 2016). As a climatic and atmospheric conditions continue to charge, the natural mechanisms that enable plants to acclimate and adjust to environmental stressors will become increasingly critical. Strengthening these protective pathways—particularly those that confer resistance to elevated UV-B radiation—may provide an effective strategy to mitigate the negative consequences of UV stress on crop productivity. Enhancing plant tolerance in this way could play a key role in sustaining agricultural output and securing global food supplies in the face of future climatic and atmospheric challenges.

Numerous reports highlight the potential of plants to remove perchlorate through phytoremediation, as they naturally uptake this compound from the soil and may accumulate it in their leaves, transform it, or transpire it (Yu et al., 2004). It is well established that perchlorate concentrations above 20 mg/L adversely affect root growth, biomass accumulation, and chlorophyll content (He et al., 2013). Moreover, even treatments as low as 5 mg/L have been shown to cause leaf damage, chlorophyll deficiency, and inhibition of photosystem II (PSII) activity in lettuce (Xie et al., 2009).

This study investigates the potential to enhance *Arabidopsis thaliana* plant UV resilience by introducing prokariotic genes identified in our laboratory through a functional metagenomics approach (Lamprecht-Grandío et al., 2020) and, in addition, potential cross-resistance to other abiotic stressors, perchlorate. These genes were isolated from microbial communities inhabiting extreme environments, including salterns in Es Trenc (Spain) and hypersaline ponds located at 4,300 and 3,200 m above sea level in the Andean Highlands of Argentina. These ecosystems are characterized by intense UV exposure, particularly in the UV-B range, making them ideal sources of UV-resistance genes.

## 2 Material and methods

### 2.1 Plant material and growth conditions

In order to obtain seeds from transgenic plants constructed using bacterial resistance genes selected from the salt flats of Diamante (located at 4,600 meters above sea level) and Ojo Seco (3,900 meters above sea level), both in Argentina, as well as from the Es Trenc salt flat (Mallorca, Spain) (Lamprecht-Grandío et al., 2020). Genes from microorganisms previously characterized for their high resistance to ultraviolet radiation were introduced into plants via *Agrobacterium*-mediated transformation. Four independent transgenic plants were obtained. pML6 *orf* 1, pML56 *orf1* and pML105 *orf* 1 contain a hypothetical protein, whereas pML56 *orf2* contain a transcription factor (Supplementary Table 1). Additionally, a wild-type *Arabidopsis thaliana* ecotype Columbia-0 was used as a control line (WT).

All seeds were surface-sterilized using 70% bleach and 0.1% Tween-20, then washed with sterile water to stratified and finally stored at 4 °C for 48-72 h. After stratification, seeds were sown on 90-mm Petri dishes containing Murashige and Skoog (MS) medium (Murashige and Skoog, 1962) supplemented with sucrose 1 % and with 0.6 % (w/w) agar, adjusted to pH 5.6. Seedlings were grown under controlled conditions 22 °C and dark photoperiod (16 h light/8 h darkness).

For soil-growning plants, seeds were stratified at 4 °C for 2-3 days prior to sowing in a 1:1 soil:vermiculite mixture or were transplanted into the same substrate after being selected from the BASTA resistance plates. These plants were maintained under the same environmental conditions, temperature and photo pheriod as those grown on MS medium

### 2.2 Plants transformation

*E. coli* DH10B strains harboring the UV-resistance genes *pML6 orf* 1*, pML56 orf1, pML56 orf2*, and *pML105 orf* 1, (Supplementary Table 1; Lamprecht-Grandío et al., 2020), were cultured in LB +Ap 50 μg/ml overnight. After incubation, plasmid DNA was isolated using QIAprep Spin Miniprep Kit. Plasmid DNA was used as template for UV-resistance genes PCR amplification using Pfu DNA polymerase under the following conditions: initial denaturation at 95 °C for 5 min; 29 cycles of 95 °C for 45 s, 55 °C for 45 s, and 72 °C for 5 min; followed by a final extension at 72 °C for 8 min. Primer sequences are listed in Supplementary Table 2.

The amplified fragments of *pML6 orf* 1*, pML105 orf* 1, and *pML56 orf2* were digested with BamHI and EcoRI, while *pML56 orf1* was digested only with BamHI. The resulting fragments were ligated into the plant expression vector pCAMBIA3500 (ligation ratio 5:1) using T4 DNA ligase (Roche); (Ausín Pascua, 2004). The ligation products were transformed into *Escherichia coli* DH5α cells using the CaCl_2_ method or electroporation when necessary. Recombinant clones were confirmed by sequencing.

The confirmed constructs (pML6 *orf* 1, pML56 *orf* 1, pML56 *orf* 2, and pML105 *orf* 1) were introduced into *Agrobacterium tumefaciens* strain C58C1 via the CaCl_2_ method (Sambrook and Russel, 2001). *Arabidopsis thaliana* ecotype Columbia (Col-0) was transformed using the floral dip method with vacuum infiltration (Clough and Bent, 1998). Given that this technique introduces the gen of interest located between the T-DNA border sequences randomly into the genome, 10 independent transgenic lines per each construct were selected based on resistance to phosphinothricin (BASTA 3 μM + 0.02 % Tween-20).

The first seeds collected from phosphinothricin-surviving, soil-grown plants following transformation correspond to the T0 generation. T0 plants were allowed to self-fertilize, and seeds were harvested and germinated on MS agar plates supplemented with 10 μg/ml BASTA. These seeds constitute the T1 generation, and subsequent generations were obtained following the same procedure. Grow T1 up for seed, seedlings that exhibited proper growth under selection were first transferred to MS agar plates and subsequently to soil, the seeds will be the next generation. T_2_ generation plants were screened as described for the T0 generation, and the best-performing lines were selected. Homozygous T3 lines were obtained for downstream experiments. The names of the selected T3 transgenic plant lines have been simplified in Supplementary Table 3. From this point onward, we will use the simplified nomenclature to facilitate readability throughout the paper.

### 2.3 RNA extraction and cDNA synthesis

**Figure 1:**
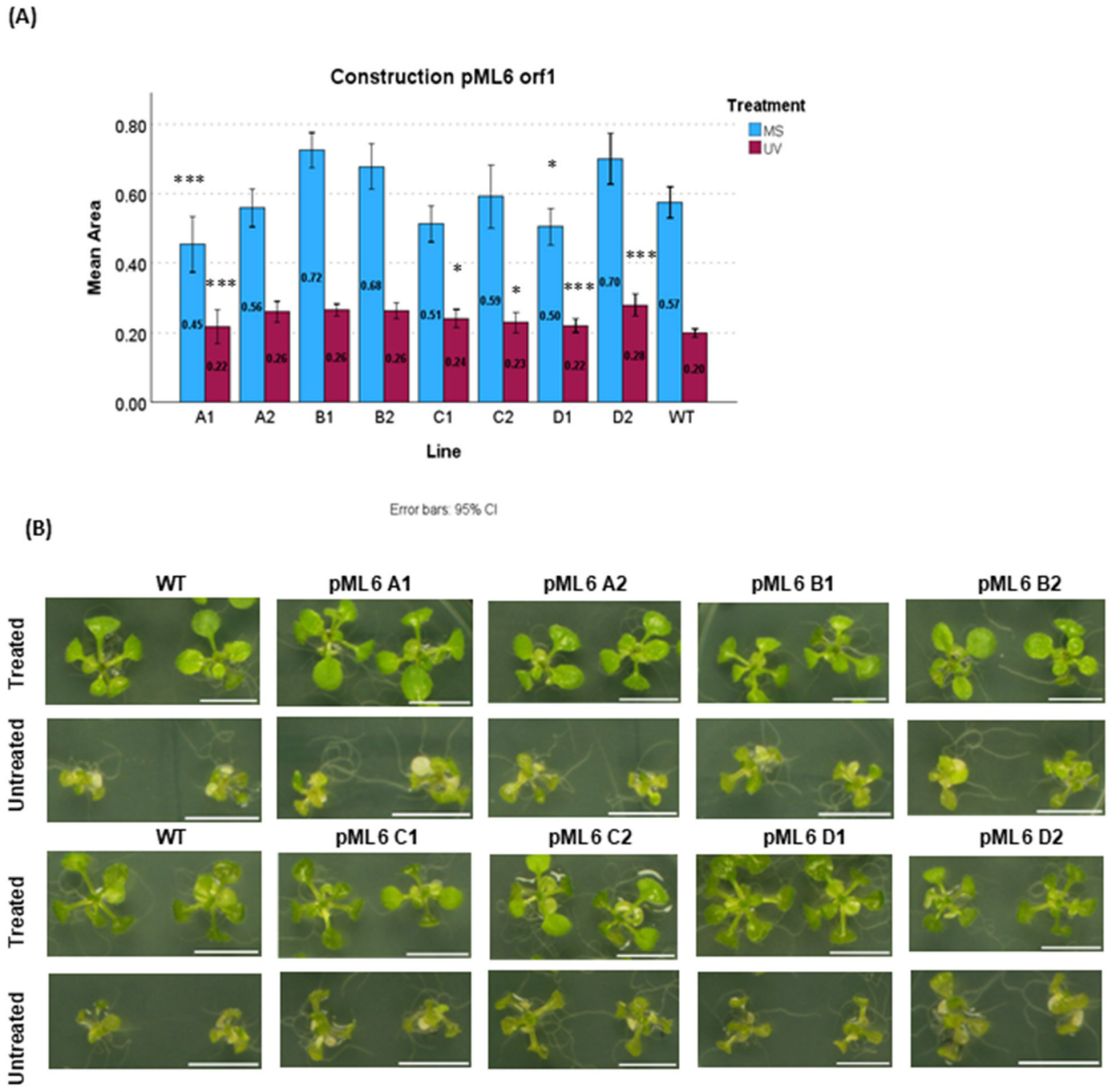
UV-C radiation resistance assay of the lines belonging to pML6 orf1 plants.. Plants were grown under a 16/8 h light/dark photoperiod on MS medium supplemented with sucrose **(A)** control plates (untreated) grown in MS medium for 15 days. Treated plates were grown for one week, treated with UV-C (4.5kJ·m−2), and then grown for an additional week under controlled conditions. A linear mixed-effects model was used to assess differences between each line vs. WT, with statistically significant differences indicated by asterisks (*P < 0.05, **P < 0.01, ***P < 0.001). Data represent the mean Rosette Area of all biological replication. **(B)** Phenotype of all selected independent transgenic plant lines pML6 orf1, after 14 days of growth (4.5 kJ·m^−2^ UV exposure). Scale bar for all images 1cm.

Total RNA was extracted, from plant tissues growing in UV stress condition, using the FastRNA Pro Green Kit (MP Biomedicals), following the manufacturer's instructions. RNA concentration and purity were assessed spectrophotometrically, and integrity was confirmed by electrophoresis on a 1 % agarose gel in TAE buffer (Supplemented Figure 1).

To remove genomic DNA contamination, 3 μg of total RNA were treated with DNase I at 37 °C for 20 min. The samples were subsequently purified through sequential extractions with phenol, phenol/chloroform, and chloroform, followed by precipitation with cold isopropanol at −80 °C for 30 min. RNA was pelleted by centrifugation, washed with 70 % ethanol, and resuspended in 8 μl of DEPC-treated water. Samples were incubated at 55 °C for 5 min and stored at −20 °C until use.

For cDNA synthesis, 8 μl of purified RNA were denatured at 65 °C for 5 min in the presence of 1 μl oligo(dT) primers (Sigma) and 10 mM dNTPs. After chilling on ice, the following components were added: 2 μl 5 × RT buffer (Invitrogen), 4 μl 25 mM MgCl_2_ (Invitrogen), 2 μl 0.1 M DTT, 1 μl RNaseOUT (Invitrogen), and 1 μl SuperScript II reverse transcriptase (200 U/μl, Invitrogen). The 20 μl reaction was incubated at 42 °C for 2 h, followed by 15 minutes at 70 °C. After chilling on ice, samples were treated with RNase H at 37 °C for 20 min.

### 2.4 RT-PCR analysis

To verify transgene expression, cDNA synthesized from RNA samples was used as a template for PCR amplification. Specific primers for each construct are listed in Supplementary Table 2.

PCR reactions were performed in 0.2 ml microtubes using PCR Core reagents (Invitrogen). Each 50 μl reaction contained 2 μl of cDNA, 2 μl of each primer (5 μM), and 0.5 μl Taq DNA polymerase (Invitrogen). The thermal cycling conditions were as follows: initial denaturation at 95 °C for 2 min; 35 cycles of 95 °C for 45 s, 55 °C for 45 s, and 72 °C for 2 min; followed by a final extension at 72 °C for 10 min.

### 2.5 Stress treatment

#### 2.5.1 UV-B/UV-C

Initially, wild-type *Arabidopsis thaliana* (Col-0) seedlings grown for 1 week on MS agar plates were exposed to UV-B (312 nm at 2.26 W·m^−2^) or UV-C radiation (254nm at 1.56W·m^−2^) using a Bio-Rad GS Gene Linker UV chamber (dimensions: 31.7 × 24.1 × 15.2 cm). Prior to irradiation, the chamber was sterilized and the UV lamp preheated for 180 s in both cases.

Plants were exposed to UV-B for varying durations of 5 min., 15min., 30min., 1 h and 2 h these exposure times corresponded to 0.68 kJ·m^−2^, 2.03 kJ·m^−2^, 4.07 kJ·m^−2^, 8.14 kJ·m^−2^ and 16.27 kJ·m^−2^ Plants were exposed to UV-C for varying durations of 1 min, 2 min 30 s, 3 min, 3 min 30 s and 4 min. these exposure times corresponded to 1.45 kJ·m^−2^, 3.29 kJ·m^−2^, 4.81 kJ·m^−2^, 5.82 kJ·m^−2^, and 6.15 kJ·m^−2^. Following irradiation, seedlings were returned to a growth chamber under controlled temperature and light conditions for an additional 7 days.

At the end of the post-treatment period, photographs were taken and the rosette leaf area of each plant on each plate was measured as described below. Growth differences between irradiated and non-irradiated wild-type plants were analyzed.

For subsequent experiments, 1 week-old transgenic pML lines and wild-type plants were exposed to a standardized UV-C irradiation cycle at an energy level of 4.5 kJ·m^−2^ using the same UV chamber and subsequently returned to the growth chamber for an additional 7 days. Plants were maintained under standard temperature and photoperiod conditions (22 °C, 16 h light/8 h dark photoperiod).

Each treatment was performed in triplicate, with 24 plants per line per experiment.

Plants directly exposed to UV-C radiation were subjected to the following analyses explained below: rosette area measured, Root length measurement, Germination rate assay, Chlorophyll content quantification, Anthocyanin content analysis.

#### 2.5.2 Perchlorate treatment

Surface-sterilized seeds from all transgenic lines and wild-type (Col-0) plants were sown on MS + sucrose + agar plates supplemented with 0, 3.67, 6.12 or 7.34 g/L sodium perchlorate (NaClO4). Seedlings were grown under controlled conditions for 14 days. At the end of the treatment period, plants were photographed, and rosette leaf area was measured as described below. This experiment was repeated two independent times, with 24 plants per line and per treatment.

To evaluate potential cross-resistance to perchlorate rosette area measured was measured in plants subjected to the three experimental conditions described above but chlorophyll and anthocyanin content were measured exclusively in plants grown on Murashige and Skoog (MS) medium supplemented with 3.67 g/L sodium perchlorate as explain below.

### 2.6 Phenotypic measurements

#### 2.6.1 Rosette leaf area

Each experimental condition consisted of two plates per treatment group (control and each transgenic line), with 12 plants per plate. Photographs were taken of 2 week-old plants—irradiated at the midpoint of their growth—using an Olympus DP80 camera. Rosette leaf area was quantified using the ImageJ image analysis software (ij154-win-java8). Measurements were obtained from all 24 plants per condition. This experiment was repeated three times.

#### 2.6.2 Root growth measurements

Plants were grown for 1 week under controlled light and humidity conditions, as described above, on MS medium supplemented with 1 % sucrose and 0.6 % agar, adjusted to pH 5.6. After this period, plants were irradiated with UV-C at a dose of 4.5 kJ·m^−2^. Immediately following irradiation, seedlings were transferred to fresh plates MS medium supplemented with 1 % sucrose and 0.8 % (w/w) agar, adjusted to pH 5.6 using sterile forceps. Plates were positioned vertically (perpendicular to the ground) and returned to standard growth conditions for an additional 7 days.

The point at which the root tip reached at the time of transfer was marked as time zero (t0). After 1 week of vertical growth, plates were photographed and root lengths were measured using ImageJ software. This experiment was repeated at least two different times, with 8 plants per line and treatment.

#### 2.6.3 Germination rate assay

At least 50 surface-sterilized seeds per line were sown on filter paper placed over MS + sucrose 1 % agar plates. Control plates were stratified at 4 °C for 48 h without irradiation. For the treatment groups, seeds were exposed to UV-C 5 MJ·m^−^^2^ and UV-B 1.091 kJ·m^−^^2^ using a BS-02 irradiation chamber (Opsytec Dr. Gröbel GmbH) equipped with TUV15W and G15T8E tubes, respectively.

After stratification, all plates were transferred to a growth chamber set to long-day conditions at 22 °C. Germination was assessed at 5 and 10 days post-stratification (Weigel and Glazebrook, 2002) Germination rate was calculated as the percentage of seeds that had germinated relative to the total number sown. This experiment was performed once, with approximately 50 seeds per plate.

### 2.7 Chlorophyll quantification

In both cases, UV-C and perchlorate, the treatment protocols were identical. Eight plants (14 days old in total) per line were collected and weighed. Each sample was placed in a 1.5 ml microcentrifuge tube with three glass beads and homogenized in a thermoblock using 1 ml of 80% (v/v) acetone (Arnon, 1949). Samples were incubated at 4 °C for 12 h, then centrifuged at 2,850g for 10 min. The supernatant was collected, and its volume recorded. Appropriate dilutions were made for spectrophotometric analysis of chlorophyll content. This experiment was performed two independent times, using 8-10 plants per replicate.

### 2.8 Anthocyanin content analysis

In this analysis plants treated with UV-C and sodium perchlorate were used. Then, followed the same protocols, anthocyanin extraction was performed following the protocol of (Swain and Hillis, 1959). Fresh plant tissue was weighed and placed in microcentrifuge tubes containing 80 % methanol with 0.5 N HCl (Solution I), and incubated overnight at 4 °C with shaking.

After centrifugation at 2,850g for 10 min, the supernatant was divided into two aliquots (~500 μl each). One aliquot was used as a blank by adding 30 % H_2_O_2_ to Solution I (1:10) immediately before use. The second aliquot was mixed with 80 % methanol containing 3 N HCl (Solution III). Both samples were incubated in the dark at room temperature for 15 min. Absorbance was measured at 530 nm using a spectrophotometer.

Anthocyanin content was calculated as:


$$\begin{array}{l} Total\;anthocyanin\\ =\left({“A^{530}}^{\prime \prime }sample\;-\;“A^{530^{\prime \prime }}blank\right)/mg\;fresh\;weight \end{array}$$

This experiment was conducted two independent times, with approximately 8 plants per replicate

### 2.9 Statistical analysis

For the various tests IBM SPSS Statistics (version 30.0.0) were used.

A statistical analysis was performed to determine whether significant differences existed between transgenic lines and wild-type controls under UV-C condition (treated and untreated). Given the variability in sample size across days, Petri dishes, and treatments, a linear mixed-effects model was employed. In this model, day was treated as a random effect, while transgenic line and treatment were considered fixed effects. To validate the robustness of the model, results were compared with those obtained using the Kruskal–Wallis test, a non-parametric method suitable for datasets that may not meet the assumptions of normality or homoscedasticity.

In the case of perchlorate, due to the limited sample size and the shape of the distribution (observed in the histogram), a univariate model was applied using both parametric (ANOVA) and non-parametric (Kruskal-Wallis) approaches were applied, in which the dependent variable was the area and the factor was the line. This approach ensures that, although the data exhibit a slight deviation from normality, this does not significantly influence the results.

Finally, based on the measurements of root length, anthocyanin content, and chlorophyll levels, non-parametric tests (Mann-Whitney and Kruskal-Wallis) are performed to evaluate differences between lines within each treatment. In this analysis, treatment is considered the main factor, while “day” is excluded from the model, as it is not a fully crossed factor.

## 3 Results

### 3.1 Assessment of UV-B and UV-C dose effects on the resistance of genetically modified plants

In an effort to enhance UV resistance in plants, the plasmids carrying ORFs that confer UV resistance *pML6 orf* 1*, pML105 orf* 1*, pML56 orf1, and pML56 orf2* (Supplementary Table 1) (Lamprecht-Grandío et al., 2020) were introduced into *Arabidopsis thaliana* via the floral dipping method.

To determine appropriate UV-C and UV-B dosages for phenotypic assessment, a preliminary dose-response experiment was conducted using wild-type *Arabidopsis thaliana* Col-0 plants. At present, it is widely accepted that UV-B radiation received daily in summer is about 2-9 kJ·m^−2^ in 40° N and 40° S latitudes (Liaqat et al., 2024; McKenzie et al., 2007). This range was selected to be included in the screening. For the UV-B screening, the selected dose range started at 0.68 kJ·m^−2^ and reached 16.27 kJ·m^−2^, whereas for UV-C the applied doses were 1.45 kJ·m^−2^ and 6.15 kJ·m^−2^. In both cases, three additional intermediate points were also tested., allowing the identification of sublethal stress thresholds.

Under UV-B exposure (Supplementary Figure 2), no significant morphological differences were observed between treated and untreated plants across the first three doses tested. However, from the 1 h onward, a slight reduction in plant size became noticeable, which was confirmed after 2 h, although the overall response remained very mild.;. Similarly, UV-C first exposure dose (1.45 kJ·m^−2^) did not induce visible damage (Supplementary Figure 3). However, plants exposed to 3.29 kJ·m^−2^ and 4.81 kJ·m^−2^ of UV-C exhibited a slight reduction in rosette area. Severe growth inhibition was observed between 5.82 kJ·m^−2^ and 6.15 kJ·m^−2^, although plants remained viable. Based on these results, a UV-C dose of 4.50 kJ·m^−2^ was selected for subsequent experiments, as it induced observable stress symptoms without causing lethality

The initial screening of the four selected genes was shown in Supplementary Figure 4. For each construct, multiple independent transgenic lines were analysed to ensure that the observed phenotypes were attributable to the introduced gene rather than to positional effects, as *Agrobacterium*-mediated transformation integrates transgenes randomly into the plant genome.

All lines generated with the pML105 construct exhibited reduced growth compared with the WT, and this construct was therefore excluded from further analysis at the T_1_ generation (Supplementary Figure 4A). In contrast, all the selected pML56 *orf1*1 lines that showed significantly higher growth on the UV-treated plates were also more robust than the WT on the untreated plates (Supplementary Figure 4B). Therefore, this construct was also discarded at the T_3_ generation, as the enhanced resistance observed in these plants could not be confidently attributed to the introduced gene but might instead result from their inherently more vigorous growth.

**Figure 2:**
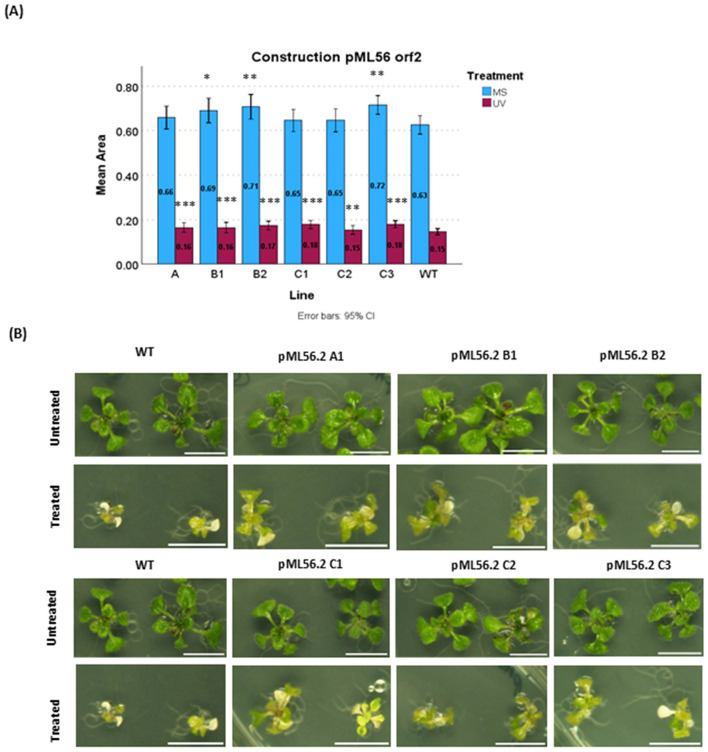
UV-C radiation resistance assay of the lines belonging to pML56 orf2 plants. Plants were grown under a 16/8 h light/dark photoperiod on MS medium supplemented with sucrose. **(A)** Control plates (untreated), grown for 15 days. Treated plates of plants were grown for 1 week, treated with UV-C (4.5 kJ·m^−2^), and then grown for another week under controlled light and temperature conditions. A linear mixed-effects model was used to assess differences between each transgenic line and wild type (WT), with statistically significant differences indicated by asterisks (*P < 0.05, **P < 0.01, ***P < 0.001). Data represent the mean rosette area from all biological replicates. **(B)** Phenotype of all selected independent transgenic plant lines pML56 orf2. Images were captured after 14 days of growth (UV exposure at 4.5 kJ·m^−2^). Scale bar for all photos 1cm.

Therefore, only the pML6 *orf* 1 and pML56 *orf* 2 lines were selected for further analysis. In both cases, at least two independent transgenic lines exhibited growth equal to or smaller than WT under non-stress conditions, yet showed significantly enhanced growth compared with WT when assessed on UV-treated plates Figures 1A, 2A.

For the pML6 *orf* 1 plants, which encodes a hypothetical protein, two independent lines were selected (D1 and A1) in the first moment (Figure 1A). It can be observed that, despite their reduced growth under control conditions, both lines demonstrated significantly improved growth under UV-C treatment compared to untreated WT plants. This suggests a potential protective effect conferred by the introduced gene under stress conditions. Additionally, other transgenic line ( C, originate from the same initial T_0_ plant but diverged at the T_1_ generation than D) does not show a statistically significant reduction compared to WT under control conditions, but exhibits similarly low growth to line D, was included in the analysis. Under UV-C exposure, this line exhibited not only significantly greater growth than WT but also outperformed the other treated transgenic lines included in the comparison. A similar behaviour was observed in another D subline (D2), which was also included. The resulting phenotype can be seen in the Figure 1B.

For the pML56 *orf* 2 plant, whose transgene encodes a transcription factor, two lines (A and C) that showed growth comparable to WT on non-treated plates were selected Figure 2A, and two additional lines from group B (lines A and B share the same T_0_ generation but differ in T_1_) and one from group C, which were slightly larger than the WT control, were included because their genomic position is identical to that of the selected lines, and the differences may be due to non-genic segregation. All selected transgenic lines demonstrated significantly improved growth relative to WT, indicating a consistent protective effect across lines. The phenotype of these plants appears in Figure 2B.

To verify that both the bacterial and archaeal transgenes were introduced into the plants and were also properly expressed, a reverse-transcription PCR (RT-PCR) analysis was performed (Supplementary Figure 1).

### 3.2 Germination efficiency and latency

In addition to rosette growth, two physiological parameters were evaluated in both transgenic and WT plants: germination percentage and germination delay. To determine the final dose to be used, germination responses were tested across a range of doses from 100 kJ·m^−2^ to 20 MJ·m^−2^. The dose of 5 MJ·m^−2^ was selected as the most suitable for obtaining clear results, which is considerably higher than the 3 kJ·m^−2^ dose established before (Rosa and Scheid, 2017). These results are summarized in Table 1, the datasets collected 5 and 10 days after UV exposure were obtained to enable the assessment of their temporal dynamics, and Supplementary Figure 5 where the final outcome can be observed after 10 days of incubation following UV treatment. The data indicate that all seeds exhibit reduced germination under UV treatment, as treated seeds consistently show lower germination percentages than untreated seeds across all lines. However, this reduction is substantially greater in wild-type (WT) seeds at 5 days. By day 10, the difference remains but becomes more comparable to the other lines. Therefore, the introduced pML56-*orf* 2 gene appears to confer protection against UV exposure, promoting and accelerating seed germination. In plants that did not receive any treatment, the transformed lines also show slightly faster and higher germination rates compared to the WT.

**Table 1:** Germination and Inhibition rate (*n* = 50) of pML56 orf2 plants.

Line	pML56 *orf*2 A	pML56 *orf*2 B1	pML56 *orf*2 B2	pML56 *orf*2 C1	pML56 *orf*2 C2	pML56 *orf*2 C3	WT
Germination percentage (%) after treatment of seeds with UV-C 5MJ/m^2^ and without treatment							
Untreated 5 days	94.44	98.04	93.94	89.19	92.105	100	88.75
Treated UV-C 5 days	90.57	93.10	94.44	83.02	86.27	92	74.65
Untreated 10 days	100	98.04	96.97	91.89	96.05	97.22	95
Treated UV-C 10 days	96.23	93.10	98.15	83.02	94.12	94	78.87
Inhibition rate (%) after treatment of seeds with UV-C 5MJ/m^2^ and without treatment							
Treated UV-C 5 days	4.11	5.03	−0.54	6.92	6.33	8	15.89
Treated UV-C 10 days	3.77	5.03	−1.215	9.655	2.01	3.31	16.975

The phenotypic observations support the numerical data, as the transformed plants exhibit a larger size and do not show the chlorosis observed in WT plants.

### 3.3 Phenotypic characterization of roots in response to UV-C radiation

**Figure 3:**
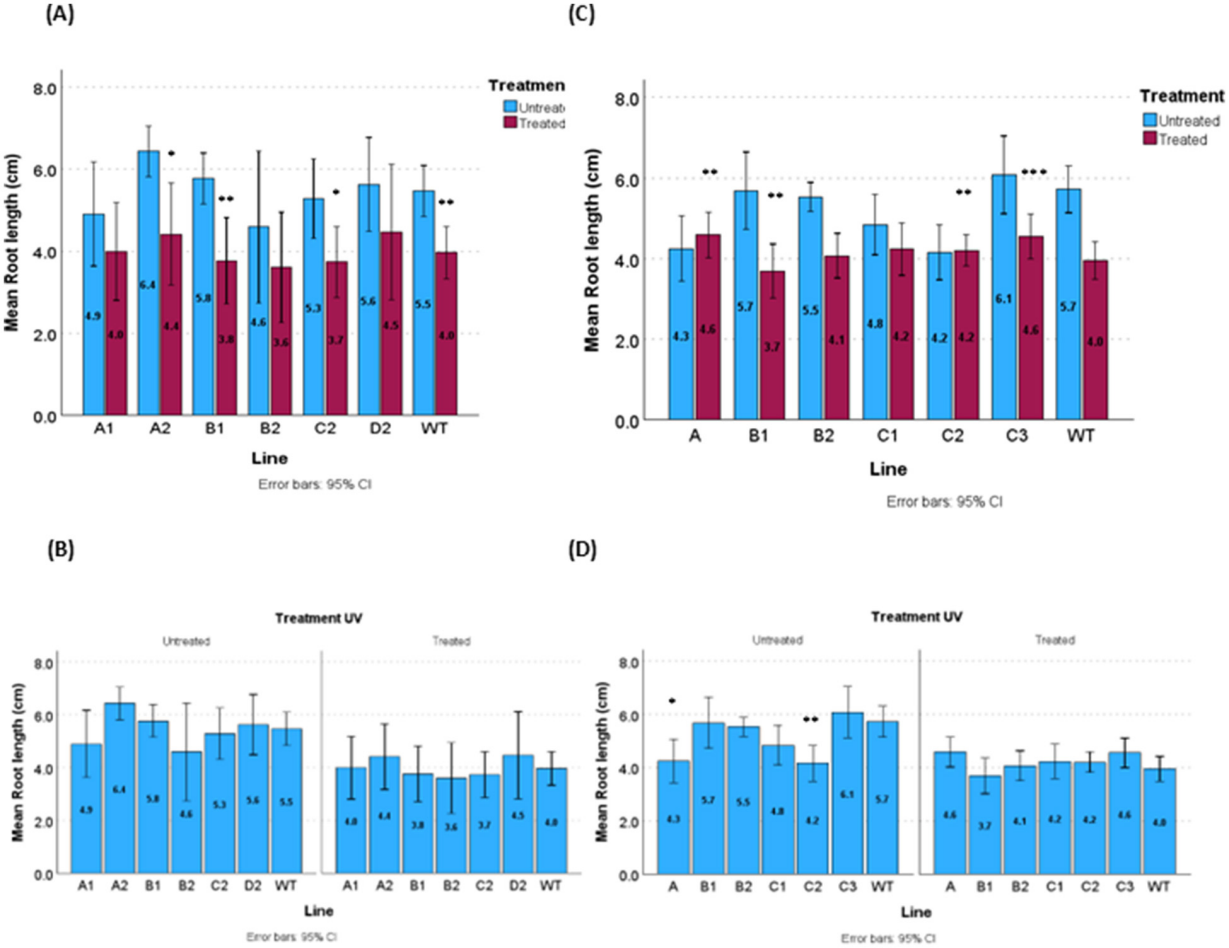
Root length of some of selected pML6 orf1 and pML56 orf2 plants (third generation, homozygous lines). 7 day-old seedling irradiated with UV (4.5kJ·m−2), following UV irradiation, plants were returned to the growth chamber for other week growing in horizontal dishes with 0.8 % agar. Bar with the average value of the lines. **(A)** Contrast of treatment vs untreated pML6 orf1 (the direction of the difference is untreated>treated UV4.5), **(B)** separate results of the average values obtained for each treatment pML6 orf1 vs WT, **(C)**. Contrast of treatment vs untreated pML56 orf2 (the direction of the difference is untreated> treated UV4.5) **(D)** separate results of the average values obtained for each treatment pML56 orf2 vs WT. The significant differences are indicated by asterisks (Mann-Whitney test, *P < 0.05, **P < 0.01, ***P < 0.001).

In parallel, vertical growth assays were conducted to evaluate root development in selected transgenic lines compared to wild-type (WT) plants under both control and UV-C stress conditions. Primary roots were allowed to grow for 7 days on Murashige and Skoog (MS) medium. At day 7, seedlings were exposed to 4,5 kJ·m^−2^ of UV-C radiation and subsequently transferred to vertical MS agar plates (0.8 %) for an additional 7 days of growth. As shown in Figure 3 and Supplementary Table 4, root length under non-irradiated conditions was comparable across all genotypes.

Nevertheless, following UV-C treatment, all transgenic lines exhibited showed a bit longer roots in each experiment but the differences were not statistically significant due to variability in the data across replicates and lines, nevertheless, in general, less loss of length than primary roots of WT plants were obtained.

### 3.4 Cross-resistance assay: response to perchlorate exposure

**Figure 4:**
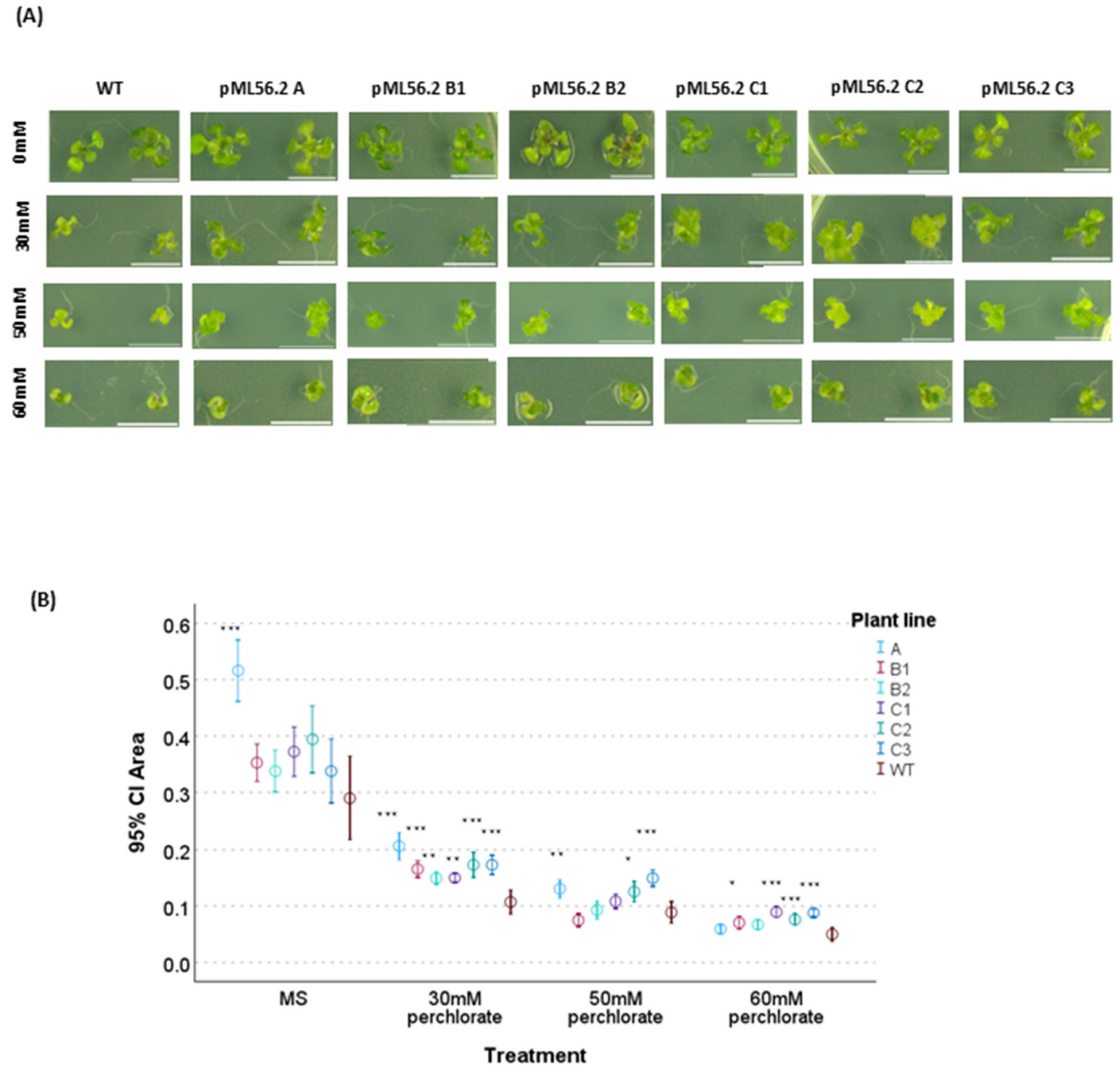
Phenotype of all selected independent transgenic plant lines pML56 orf2 (third generation, homozygous lines). **(A)** Images were captured after 14 days of growth on MS medium supplemented with sucrose, including both control (untreated) and treated samples (plates grown in MS for 15 days with 0, 3.67, 6.12 and 7.34 g/L sodium perchlorate). **(B)** Effects of perchlorate in plants. Mean values of rosette area for the six lines selected from pML56 orf2 [two independent transgenic lines (A and C), along with a variant of line A (B)], transformation after 15 days growth in different concentration of sodium perchlorate MS medium plates. The significant differences are indicated by asterisks (Bonferroni test and Dunnett, *P < 0.05, **P < 0.01, ***P < 0.001). Scale bar for all photos 1cm.

The genes selected for UV resistance also conferred resistance to perchlorate—up to three orders of magnitude—in laboratory assays in which additional stress conditions were evaluated (Lamprecht-Grandío et al., 2020). For this reason, resistance tests using this compound were also performed on the plants transformed with these genes. Growth differences between transgenic lines and wild-type (WT) plants were assessed after 15 days of treatment (Figure 4A), Sodium perchlorate was used in all experiments, as it is less toxic than ammonium perchlorate yet has proven to be a useful tool for elucidating the specific effects of the perchlorate anion (${\text{ClO}}_{4}^{-}$) (Heinz et al., 2019). All perchlorate concentrations tested affected the plant phenotype, as the rosette leaves of all treated plants appeared somewhat shorter, more rigid, and less spread out. Initially, these phenotypic differences were not very noticeable; however, after approximately 1 month they became much more evident, resembling the vitrification symptoms observed in plants exposed to excessive humidity. At a concentration of 3.67 g/L sodium perchlorate, all transgenic lines carrying the pML56 *orf2* construct exhibited significantly greater growth than WT. At higher concentrations (6.12 and 7.34 g/L, a similar trend was observed; however, increased variability among some lines reduced the statistical significance of the differences. Nonetheless, at both concentrations, at least two transgenic lines showed significantly improved growth compared to WT (Figure 4B).

The same experiment was attempted with the pML6 *orf* 1 construct, but seed viability declined during the assay, and the results were inconclusive regarding differential resistance to perchlorate across lines (data not shown).

### 3.5 Evaluation of plant development in soil substrate

**Figure 5:**
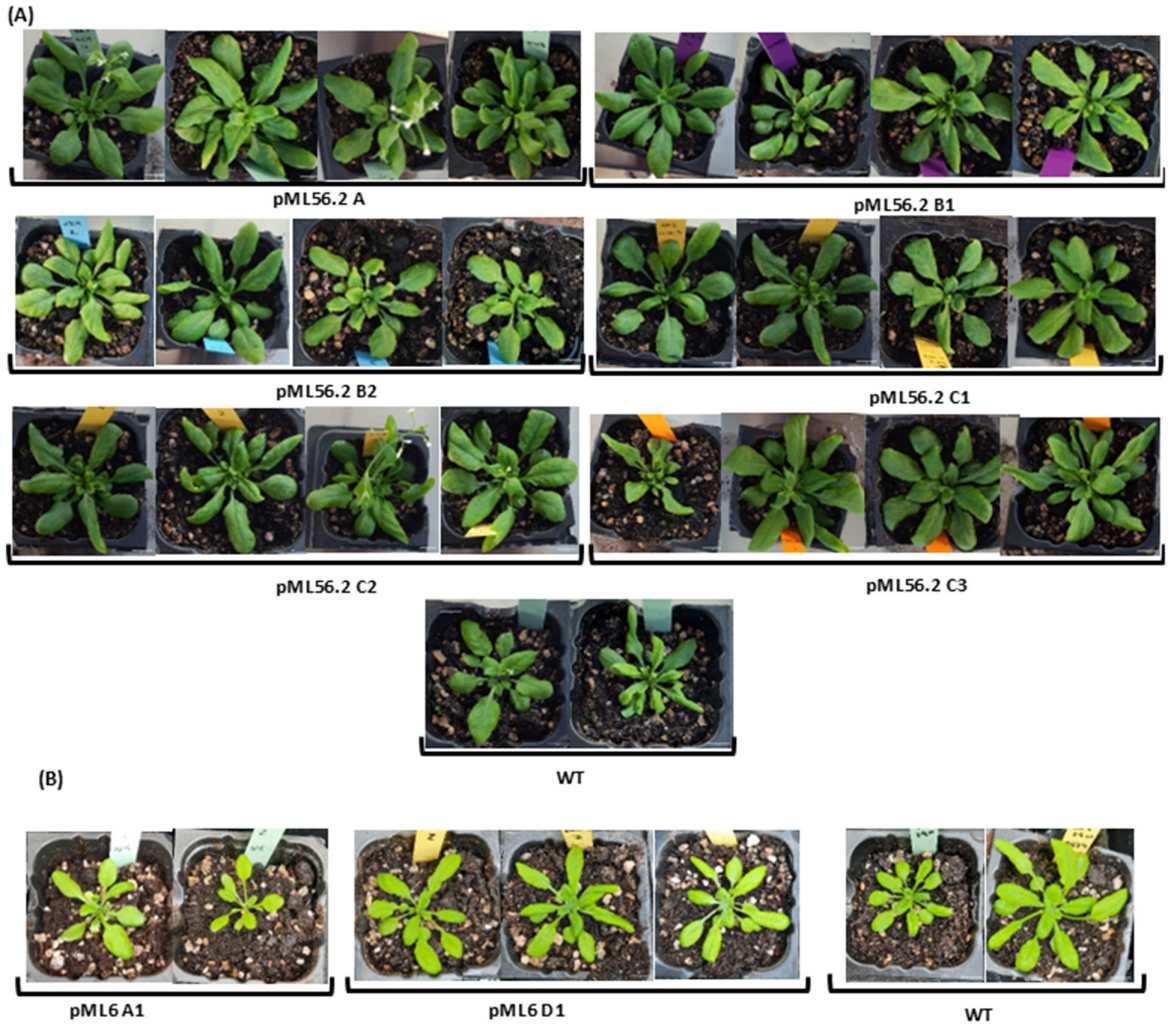
Phenotypes of *Arabidopsis thaliana* plants in soil. **(A)** selected lines of pML56 orf2 **(B)** pML6 orf1. Scale bar for all photos 1 cm.

Selected lines from the pML56 *orf* 2 and pML6 *orf* 1 constructs were grown to the T_3_ generation for phenotypic evaluation (Figure 5). Throughout vegetative development—including cotyledon emergence, early leaf formation, and subsequent growth stages—no visible morphological abnormalities or deviations from wild-type (WT) plants were observed. All transgenic lines tested showed confirmed expression of the introduced genes (Supplementary Figure 1). Not obvious structural abnormalities were detected in our phenotyping set-up. These observations suggest that the expression of both the hypothetical protein (pML6 *orf* 1) and the transcription factor (pML56 *orf* 2) did not interfere with normal vegetative development. Plants grown in soil also showed no signs of altered rosette morphology, such as leaf curling toward the abaxial surface or delayed flowering time, although there do appear to be other phenotypic defects related to leaf size, leaf number, and earliness, which suggests that more comprehensive phenotyping would likely be necessary to accurately assess the actual impact of these genes on the plant phenotype.

### 3.6 Chlorophyll and anthocyanin quantification as indicators of UV- and perchlorate- induced stress

**Figure 6:**
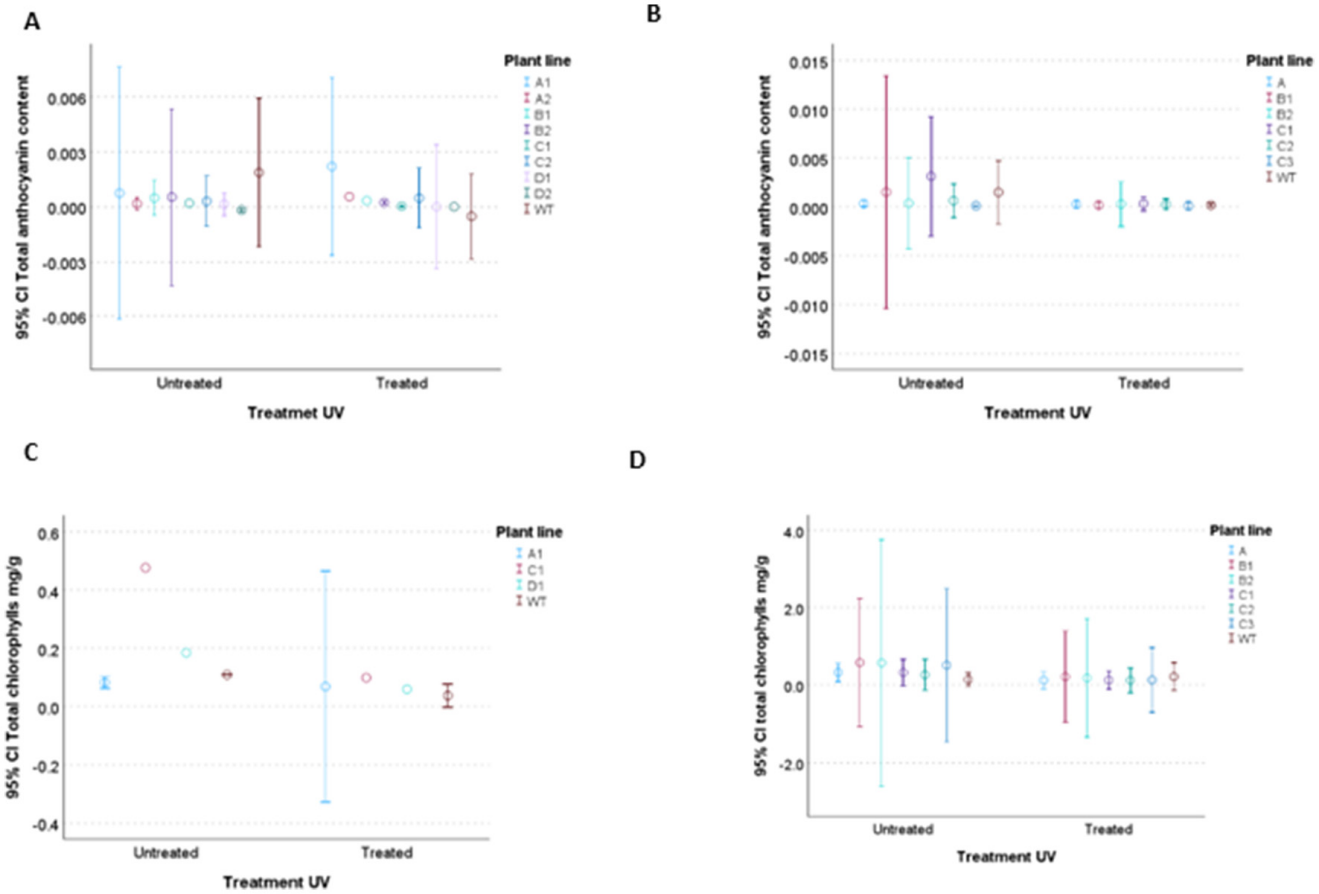
Effects of UV radiation on the chlorophyll and anthocyanin content in plants selected. **(A)** Total anthocyanin content in plant with pML6 orf1, **(B)** total anthocyanin content in plant with pML56 orf2, **(C)**. total chlorophylls in plant with pML6 orf1, **(D)** total chlorophylls in plant with pML56 orf2. Error bars represent 95 % confidence intervals (95 % CI). Non-overlapping CIs suggest statistically significant differences between groups.

Additionally, chlorophyll and anthocyanin content were quantified in all lines 7 days after UV-C irradiation, as well as in non-irradiated control conditions. The results of these biochemical analyses are presented in Figure 6. As shown in the graphs, the anthocyanin levels in untreated and UV-treated plants did not exhibit major differences between treatments, nor did they show statistically significant differences between the transformed plants and WT. In both constructions, however, WT showed a greater decrease in anthocyanin content in the treated plants compared with transformed plant, likely because in untreated experiments it starts with a higher basal anthocyanin concentration. A similar pattern was observed for chlorophyll content: all tested plants showed a decrease in chlorophyll when exposed to UV, but the differences relative to the WT were not statistically significant in any case, even if the plants carrying the pML6-*orf* 1 gene appeared to perform slightly better than the WT.

## 4 Discussion

Previous studies in plants have shown that ultraviolet (UV) radiation reduce growth of plants and can cause permanent damage or even death if it is out of their tolerance limits (Liaqat et al., 2024; Mosa et al., 2017). This damage is particularly localized in the DNA (Rastogi et al., 2010; Sinha and Häder, 2002), as well as proteins and lipids (Kataria, 2017; Kumari et al., 2022; Prinsze et al., 1990; Smirnoff, 1995). The induction of genetic damage is considered a primary target of UV stress in plants and other organisms (Quaite et al., 1992; Tuteja et al., 2009). It is well established that the effects of UV radiation vary significantly among plant species (Teramura and Sullivan, 1994) dicotyledons plants are more susceptible to UV-B radiation than monocotyledons (Kataria, 2017), and even among ecotypes within the same species (Shishkin and Ivanishchev, 1997; Torabinejad and Caldwell, 2000). Notable differences in light response have also been reported (Cooley et al., 2001; Maloof et al., 2001) demonstrating a correlation between growth rate and UV-B sensitivity across different *Arabidopsis* ecotypes. In this study, we used the Col-0 ecotype of *Arabidopsis thaliana*, which is a dicotyledonous plant, and has been shown to exhibit an intermediate transcriptional response to UV-B stress-related genes (Kalbina and Strid, 2006). This makes Col-0 a suitable model for evaluating the effects of UV-induced stress and the potential protective role of introduced genes. The floral dipping technique with *Agrobacterium* was used, whose main advantage is that the introduced genes will be stably integrated into the plant genome and can be expressed (Clough and Bent, 1998). The resulting proteins could enhance the plant's ability to defend itself against UV radiation.

A potential limitation of this approach is the introduction of bacterial and archaeal genes, which can occasionally hinder proper protein expression in eukaryotic hosts due to differences in codon usage, regulatory elements, and post-translational modification requirements (Parvathy et al., 2022). Despite these constraints, expression of the heterologous genes was confirmed in all selected transgenic lines prior to conducting the resistance assays.

Another drawback was the extended exposure time required for the UV-treatment assays, which was constrained by the large number of transgenic lines generated per construct and processed simultaneously. It is known that plants respond to UV-B light, regulated not only by the wavelength intensity but also by its duration (Chen et al., 2022). Although UV-B is naturally present in the environment and biologically relevant and can inhibit growth through cellular damage (Jansen et al., 2012), UV-C—due to its shorter wavelength and higher photon energy—induces comparable damage in a significantly shorter exposure time; (Çavuşoglu et al., 2022). While UV-B exposure has been shown to significantly influence plant growth and development, prolonged UV-C exposure induces physiological stress responses (Jazayeri et al., 2024). According to Planck's relation, shorter wavelengths correspond to higher photon energy; thus, UV-C radiation has been shown to cause DNA damage at levels approximately 106 times greater than UV-A (Banaś et al., 2020). Furthermore, it was taken into consideration that UV-B and UV-C share the same receptors and signalling pathways, and both radiations seem to be the origin of similar effects. Even if this does not exclude the possibility that UV-C radiation may exert additional specific effects beyond those currently identified (Urban et al., 2016). Based on the results obtained from both UV-B and UV-C laboratory assays, UV-C radiation was selected for subsequent experiments. A dose of 4.5 kJ·m-^2^ was selected, which is more than twice the dose previously established as genotoxic and stress-inducing (2 kJ·m-^2^) (Mintoff et al., 2015) to explore the maximal tolerances of our experimental models. The decision to use UV-C instead of UV-B allowed us to shorten the irradiation time to approximately 3 min per plate, whereas UV-B treatments typically require exposures of 2 h or longer.

Quantitative analyses revealed that transgenic plants containing the selected resistance genes exhibited reduced damage under UV-C stress compared to wild-type controls. This was evident in parameters such as rosette size and germination rate, which are known to be negatively affected by UV-C exposure (Hernandez-Aguilar et al., 2021; Pournavab et al., 2019; Sarghein et al., 2011). A particularly noteworthy finding from this study was the identification of a hypothetical protein encoded by pML6 *orf* 1, contained in plasmid pML6, which not only conferred UV resistance but also appeared to enhance overall plant resilience. This gene had previously been proposed by Lamprecht-Grandío et al. (2020) as being involved in DNA damage repair, and our results provide further support for its functional role in stress mitigation.

The pML56 plasmid contained two distinct open reading frames (ORFs), each of which was independently introduced into plants, resulting in different phenotypic outcomes. pML56 *orf* 1, which encodes a Hypothetical protein, exhibited a generally more robust phenotype than WT in untreated plates in those lines that also exhibited significantly enhanced growth under UV exposure, suggesting a general growth alteration unrelated to UV resistance. Given that the observed phenotype may not be attributable solely to the functional activity of the introduced gene, but rather to positional effects or unintended genomic alterations associated with the transformation process, this construct was ultimately excluded from further analysis. In contrast, this study demonstrates enhanced UV-C resistance in *Arabidopsis thaliana* plants transformed with the pML56 *orf* 2 gene, which encodes a TATA-box binding protein (TBP) from the archaeon *Haloferax volcanii*. The introduction of this gene not only improved plant growth under UV-C stress but also mitigated its typical stress-related effects, such as reduced germination rate, growth retardation, and inhibition of development, effects commonly observed in wild-type plants exposed to UV-C (Kalbina and Strid, 2006; Pournavab et al., 2019). In fact, transformed plants germinated following UV-C seed exposure showed less chlorosis compared with the WT ones (Supplementary Figure 5). Notably, no morphological abnormalities were observed in the leaves or rosette structure of the transgenic plants, in contrast to the phenotypic alterations reported before (Li et al., 2001) when overexpressing a TBP gene, which severely affected aerial vegetative development (Figure 5). The findings presented here support previous suggestions by Lamprecht-Grandío et al. (2020) that this gene may help the organism to survive in an extreme environment, even if it cannot be assured a role in DNA protection or repair. Although previous studies have already proposed that TBPs are involved in transcription-coupled repair mechanisms, facilitating the removal of DNA lesions from actively transcribed genes. facilitating the removal of DNA lesions from actively transcribed genes (Balajee and Bohr, 2000). Furthermore, transcriptomic analyses have shown that approximately 60% of oxidative stress-responsive genes in *Arabidopsis thaliana* contain a TATA box in their promoter regions, compared to only 20 % of genes related to protein folding (Zhang et al., 2017). More recently, Haga clic o pulse aquí para escribir texto (Savinkova et al., 2023) proposed that the TATA box is indirectly involved in the regulation of light-responsive gene expression, suggesting that increasing TATA box availability could enhance plant adaptability to environmental stressors.

However, we were able to successfully perform growth assays in the presence of perchlorate, given that the selected genes conferred protection against DNA damage in transformed bacteria (Lamprecht-Grandío et al., 2020). It is possible that, in plants, this function is likewise associated with DNA protection. However, an interesting finding derived from this study was that the plants transformed with the pML56 *orf* 2 gene exhibited not only enhanced resistance to UV radiation but also cross-resistance to perchlorate, a compound that elicits oxidative stress. This compound is also of particular interest because it occurs on Earth as a soil contaminant (Seyfferth and Parker, 2008), and also on Mars it is associated with the depression of the freezing point of water and with the formation of liquid water due to its strong hygroscopic capacity (Zorzano et al., 2009). It is well established that many plant species can absorb perchlorate from soil or water (Susarla et al., 1999); and tends it to accumulate in leafy tissue thanks to transpiration. Nevertheless, plants do not possess the ability to hyper accumulate it, once their uptake capacity is exceeded, plants may begin to exude, transform, or transpire perchlorate (Yu et al., 2004) or phytodegrade (Nzengung et al., 2004; Susarla et al., 2000). Although the precise mechanisms by which perchlorate affects plant physiology remain unclear, it is known to particularly impact leaf tissues and the oxidative capacity of roots—both of which are highly sensitive to this compound. Perchlorate is known to be involved in the active co-transport processes in plants, with uptake efficiency decreasing as pH increases (Cataldo et al., 1986; Hurd, 1958). However, the experiments conducted in this study were performed at a pH of 5.6. Perchlorate exposure has been shown to alter chlorophyll content and reduce the efficiency of photosystem II (PSII), leading to varying degrees of inhibition and damage in the photosynthetic apparatus and leaf structure (Xie et al., 2009). Previous studies have shown that treatment with 500 mg/L of sodium perchlorate leads to a significant reduction in chlorophyll content in plant leaves compared to untreated controls, likely due to the compound's oxidative properties (He et al., 2013).

In our study, transgenic plants carrying the pML56 *orf* 2 construct (encoding a TATA-box binding protein) exhibited enhanced growth under perchlorate stress, even at the lowest concentration tested 3.67g/L with respect to wild-type (WT) controls under the same conditions. However, a deviation from the typical rosette morphology was observed, with leaves displaying a more disorganized growth pattern progressively more pronounced over time. This remarkable phenotype may be related with the presence of perchlorate and to the excess moisture generated either within the plant tissues or on the culture plate.

Previous studies have shown that exposure to UV-C and UV-B radiation causes chloroplast damage in *Arabidopsis thaliana* and reduces the expression of several key photosynthetic genes (Çavuşoglu et al., 2021; Urban et al., 2016; Wituszynska et al., 2015; Yadav et al., 2020). In addition, this radiation inhibits photosystem II (PSII) activity, primarily by disrupting the lamellar membrane structure (Mantai et al., 1970) and, secondarily, by promoting the degradation of plastoquinones (Bishop, 1961). It also leads to a reduction in chlorophyll content (Klem et al., 2015). Despite the introduction of resistance genes, chlorophyll quantification assays did not show significant differences compared with the control. This suggests that the introduced genes may not effectively preserve photosynthetic function. However, they could still confer protection against UV- and/or perchlorate-induced oxidative stress, albeit not at the level of maintaining PSII function.

On the other hand, UV-C radiation has been shown to stimulate an increase in phenolic compound accumulation (Nigro et al., 2000), notably enhancing the biosynthesis of flavonoids, including anthocyanins (Eichhorn Bilodeau et al., 2019; Liu et al., 2020). These secondary metabolites possess antioxidant properties and play a protective role against UV-induced oxidative stress (Rivera-Pastrana et al., 2014), even though the role of anthocyanins in UV-B protection remains unclear due to existing controversy. Some studies proposed that anthocyanins do not contribute to the UV-B protective system (Hada et al., 2003), contradicting other findings that reported a cooperative role of anthocyanins in such protective mechanisms (Gitz et al., 1998). However, the total anthocyanin content was quantified in plants treated with UV-C to evaluate the physiological impact of gene introduction. The results revealed that transgenic lines, particularly those carrying pML6 *orf* 1, retained slightly higher levels of both pigments compared to wild-type controls under UV-C stress, and, in the case of pML56 *orf* 2 its behaviour is similar to WT with and without treatment. This suggests that the introduced genes may not contribute to overproduction of flavonoids. It is also possible that the results obtained could be influenced by the specific treatment parameters, as UV-C-induced responses have been shown to be highly dependent on treatment conditions (Pontin et al., 2010). It is known than flavonoid accumulation began to increase at 48h with UV-A, 24h with UV-B but, a duration of only a few min may not be enough with the high UV-C dose (Hollósy, 2002).

## 5 Conclusion

The introduction of the prokariotic genes pML6 *orf* 1 and pML56 *orf* 2, which encode a hypothetical protein and a TATA-box-binding protein respectively, into the model plant *Arabidopsis thaliana* has been shown to confer enhanced resistance to UV-C radiation—the most harmful form of ultraviolet radiation. Transgenic plants exhibited significantly improved growth and accelerated germination rates compared to non-transformed controls under UV-C exposure.

Chlorophyll and anthocyanin quantification revealed that the enhanced UV-C resistance is not associated with increased protection of chloroplasts or photosynthetic efficiency, nor with the accumulation of typical UV-protective secondary metabolites such as phenolic compounds.

Notably, the construct containing the TATA-box-binding protein also conferred cross-resistance to sodium perchlorate, an inducer of other oxidative stress. Transgenic plants were able to survive on media containing 3.67, 6.12, and even 7.34 g/L of perchlorate concentrations significantly higher than those previously reported in the literature (He et al., 2013).

These findings suggest that the introduction of such genes into plants may open new avenues for improving existing crops and plant species, helping to prevent declines in food production that are critical for a growing population. This is particularly relevant in the context of increased UV-B radiation at the Earth's surface. Additionally, such genetic modifications could enhance plant resistance to other oxidative stresses, such as those caused by perchlorate contamination, which currently affects many agricultural soils. Moreover, this cross-resistance could make transformed plants a good option for use in phytoremediation of areas contaminated with perchlorate.

Beyond terrestrial applications, these genetic strategies may also have astro biological relevance, potentially contributing to the development of resilient plant systems for use in extra terrestrial environments. It has already been suggested that perchlorate resistance is essential for potential microbial life on Mars, as perchlorate has been present on the planet for hundreds of millions of years and is linked to water availability (Díaz-Rullo and González-Pastor, 2025; dos Santos et al., 2025). Combined with the ability to tolerate both UV-C radiation -which suggests shared resistance mechanisms- this supports the plausibility that photosynthetic organisms could endure the combined harsh radiation and perchlorate milieu on Mars or similar exoplanets, contributing to the elaboration of astrobiological habitability models.

## Data Availability

The original contributions presented in the study are included in the article/Supplementary material, further inquiries can be directed to the corresponding author.
