# Supplementary material for: Enhancing UV-C and perchlorate resistance in Arabidopsis thaliana through the introduction of microbial genes from hypersaline environment
Source: Front Microbiol. 2026 Apr 15;17:1789302. doi: 10.3389/fmicb.2026.1789302 (PMC13125036; doi:10.3389/fmicb.2026.1789302)
Supplement: Supplementary file 1 [file Data_Sheet_1.pdf]

Supplementary Material

Supplementary Figures and Tables

1.1 Supplementary Figures

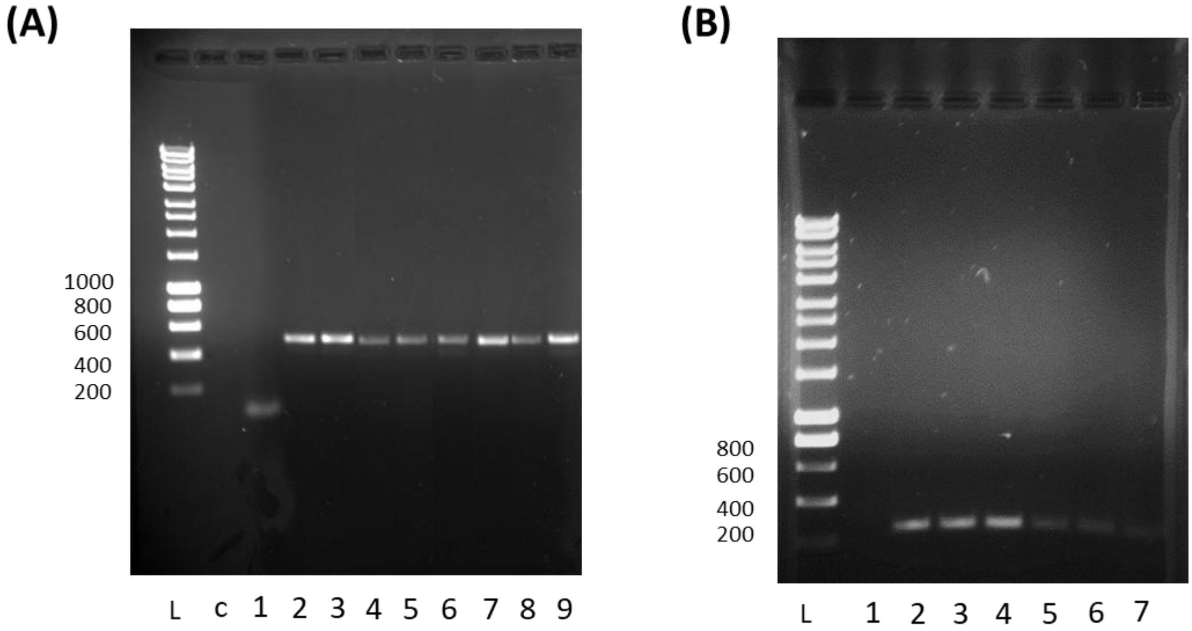

Supplementary Figure S1. Genes expression in transgenic plants. Agarose gel electrophoresis of RT-PCR products. Amplified cDNA fragments from total RNA extracted from UV treated plants were separated on a 1% agarose gel and visualized under UV light after ethidium bromide staining. L-Molecular weight marker (10.000-200pb DNA ladder); **(A)** pML6 *orf*1 T3 generation C-control with water, line 1 WT plants and plants from 8 independent transgenic lines 2-9:, A1, A2, B1, B2, C1, D1, D2, C2 (550pb). **(B)** pML56 *orf*2 T3 generation, line 1 WT plants and plants form 6 independent transgenic lines 2-7: A, B1, B2, C1, C2, C3 (310pb).

1.2 Supplementary Figures:

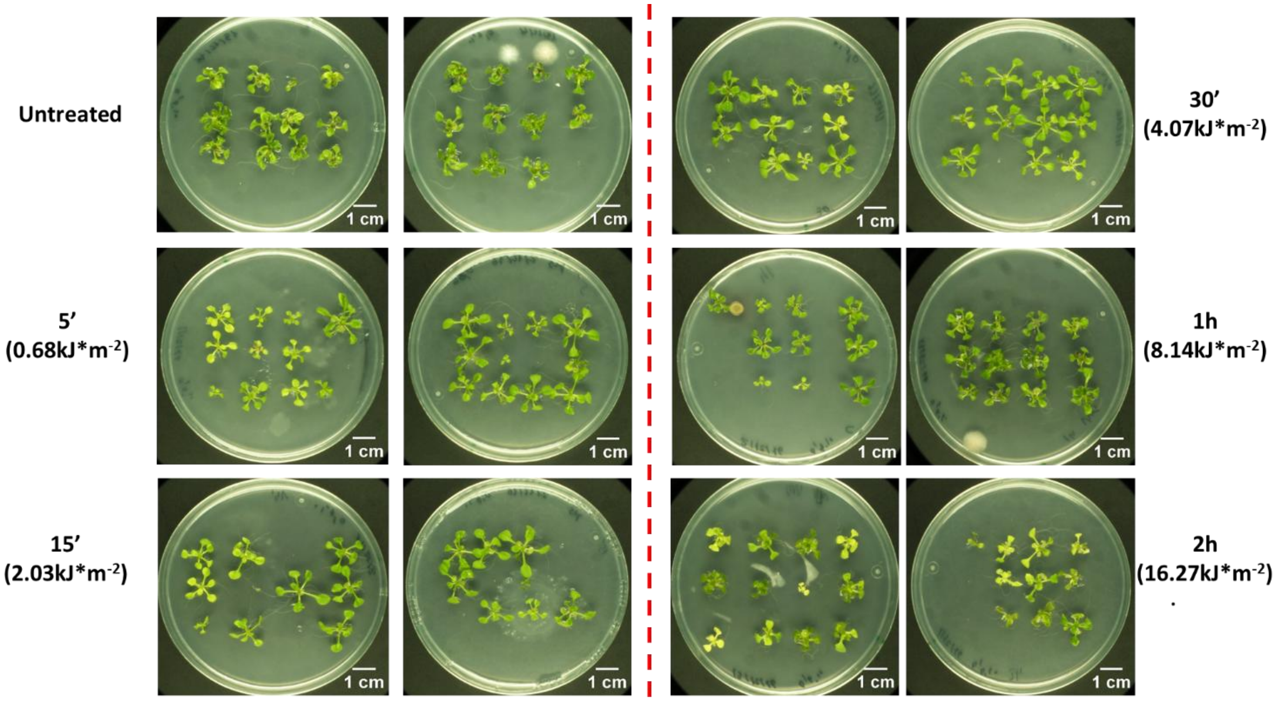

Supplementary Figure S2: Assay to select the most effective UV-B dose in *Arabidopsis thaliana* Col-0 plants. Plant phenotypic responses during the UV-B screening. Seedlings were cultivated for 7 days under controlled environmental conditions in a growth chamber (temperature, photoperiod, and humidity maintained as specified). Plants were then exposed to graded UV-B doses. Following irradiation, they were returned to the growth chamber and allowed to recover for another 7 days before phenotypic assessment. Scale bar for all photos 1cm.

1.3 Supplementary Figures:

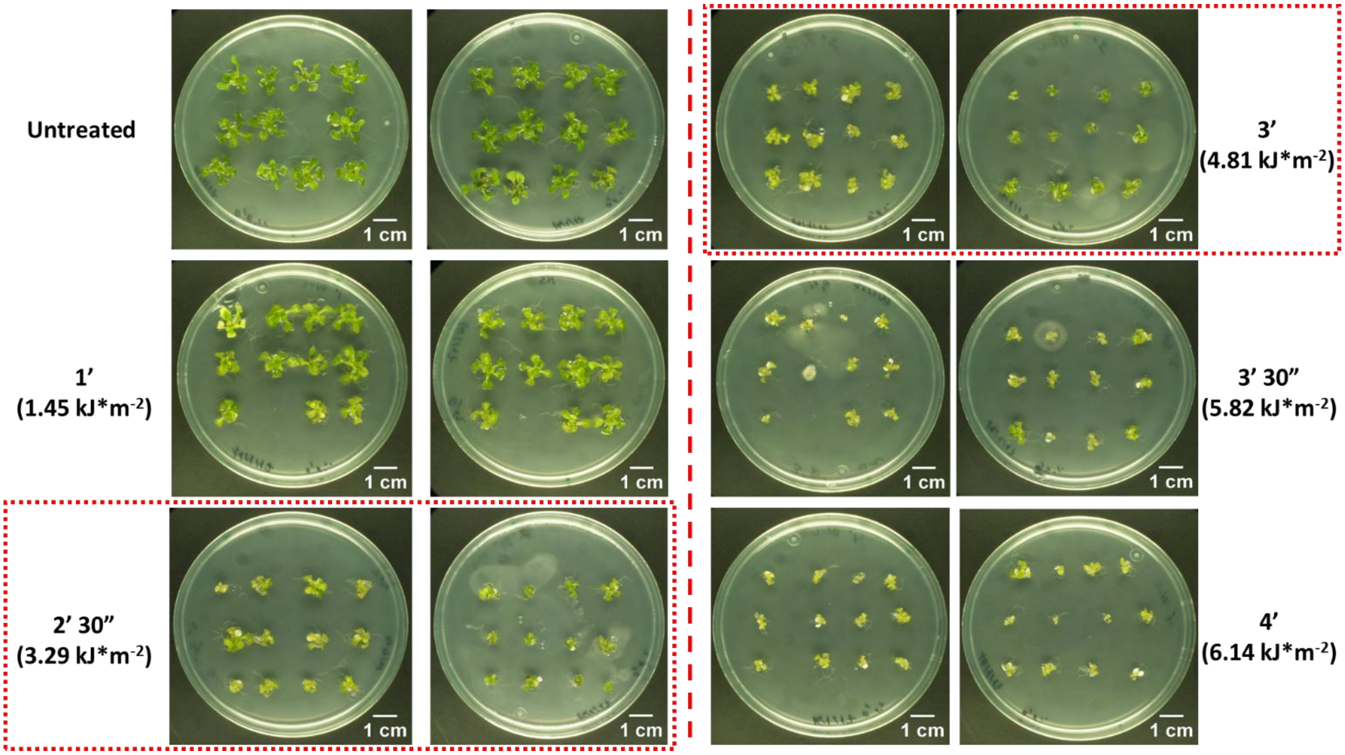

Supplementary Figure S3: Assay to select the most effective UV-C dose in *Arabidopsis thaliana* Col 0 plants. Plant phenotypic responses during the UV-C screening. Seedlings were cultivated for 7 days under controlled environmental conditions in a growth chamber (temperature, photoperiod, and humidity maintained as specified). Plants were then exposed to graded UV-C doses. Following irradiation, they were returned to the growth chamber and allowed to recover for another 7 days before phenotypic assessment. The red dotted line marks the two concentrations with better results chosen for the subsequent experiments (the final experiment will be conducted using an intermediate UV-C concentration). Scale bar for all photos 1cm.

1.4 Supplementary Figures:

A

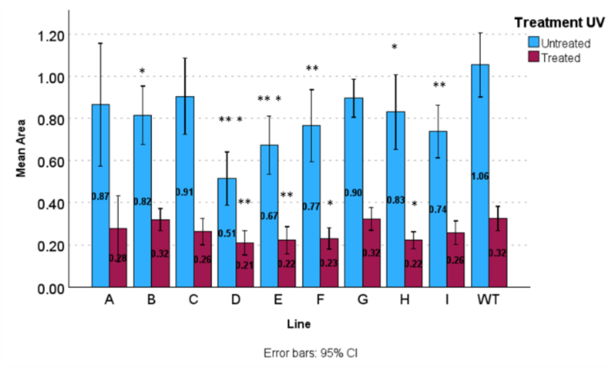

Contruction	pML105 orf1								
Original line name	6	9	10	11	12	13	14	15	16
Correpondig name	A	B	C	D	E	F	G	H	I

B

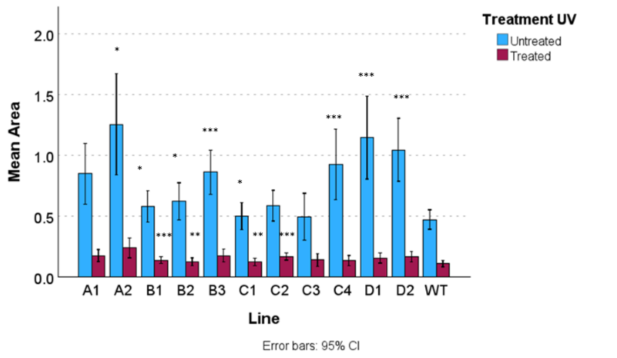

Contruction	pML56 orf1										
Original line name	8.6.1	8.7.1	9.1.1	9.8.1	9.10.1	10.1.1	10.6.1	10.7.3	10.8.1	11.5.1	11.10.1
Correpondig name	A1	A2	B1	B2	B3	C1	C2	C3	C4	D1	D2

Supplementary Figure 4: Representation of transgenic plant lines compared to wild type (WT) under control and UV treatment conditions (4.5 kJ·m^{-2}) treatment in the middle of grown. 15 days grown. **(A)** construction with pML105 gene, T$_1$ generation. **(B)** construction with pML 56 orf1 gene, T$_3$ generation. Mean Rosette Area, mixed lineal model. The significant differences are indicated by asterisk (*P<0.05, **P<0.01, ***P<0.001); both MS and UV treatment use WT as the reference for statistical comparisons.

1.5 Supplementary Figures

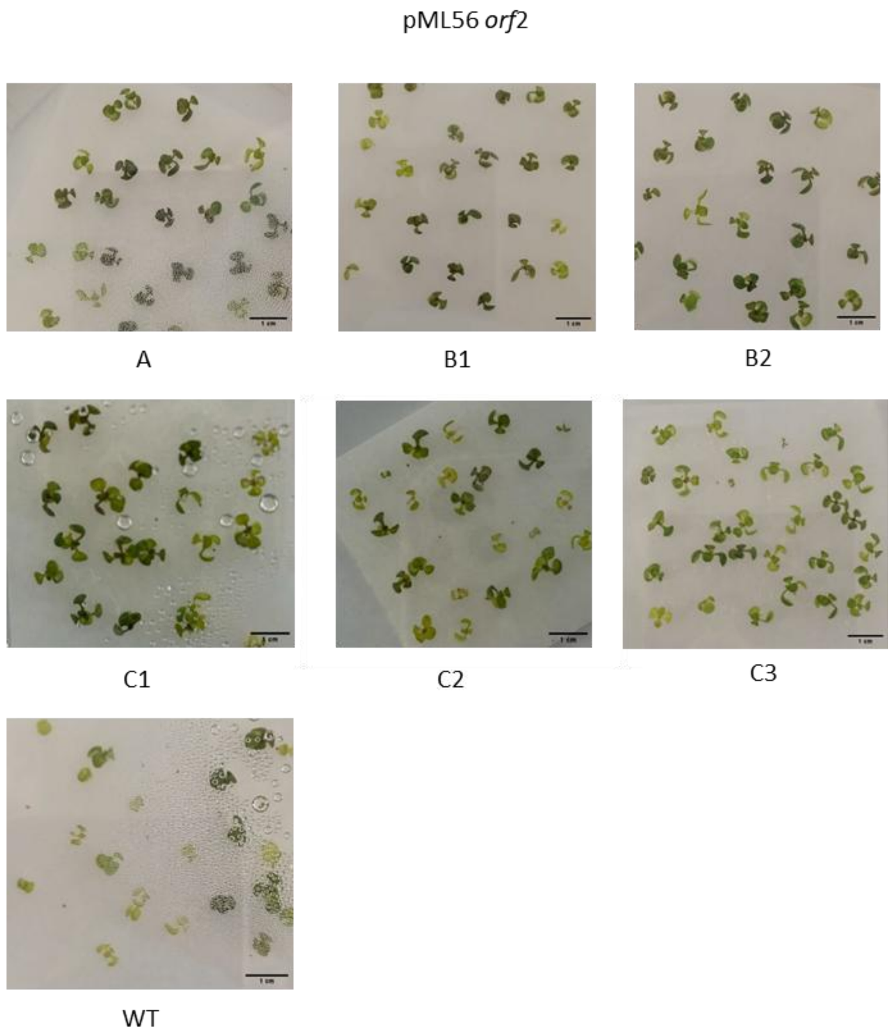

Supplementary Figure 5: Images from the germination ratio assay results of plants pML56 *orf*2 after 10 days (n=50). Seeds were treated with UV-C 5 MJ·m-2 and placed on sterile filters over MS medium plates supplemented with sucrose at time zero. They were grown for 10 days under controlled light and temperature conditions. Results were collected midway through the process (day 5) and at the end (day 10). Scale bar for all photos 1cm.

1.6 **Supplementary Table 1:** Description of the UV-resistant plasmids and their observed sequence similarities. OS: Ojo Seco, D: Diamante and PMB: Es Trenc.

**ID clones [length(bp)] % GC**	**Library of origin**	**Gene Bank accession number**	**Nº of ORFs**	**Closest similar protein (microorganism)**	**Accession number**	**Putative domain**
pML 6 (861) 46%	OS	MF495892	1*	**Conserved hypotethetical protein** (*Rikettsia* endosymbiont of *Ixodes scapularis*)	EER 22366.1	Hypothetical protein
pML 56 (812) 49%	D	MF495893	1*	**Hypothetical protein**	NA	NA
			2*	**Transcription factor** (*Haloferax volcanii*)	WP 004042154.1	TATA box binding protein
pML 105 (1547) 48%	PMB		1*	**Hypotethetical protein** (*Moritella dasanensis*)	WP 017221522.1	Hypothetical protein

Asterisks (*) indicate the ORF that provide UV the resistance (Lamprech-Grandío et al., 2020). Those sequences with an E-value higher tan 0.001 in BLASTP searchers were considered to be unknown proteines

1.7 **Supplementary Table 2:** The nucleotide sequences used for PCRs amplification of target genes

**Primer name**	**Orientation**	**Sequence (5’-> 3´)**
**pML6**	Forward	TGAGGATCCATGAATATCTTTGCCGTATC
**pML6**	Reverse	TGAGAATTCTTATCATAGCCATTCAGGCGGT
**Pml105**	Forward	TGAGGATCCATGAACATTTTTATTTAGATCA
**Pml105**	Reverse	TGAGAATTCCTATCATCCCGCGCGTCTGGA
**Pml56 *orf1***	Forward	TGAGGATCCATGTTCAATGACGCTATCAC
**Pml56 *orf1***	Reverse	TGAGGATCCTTACTAACATCTTCACCGACAAA
**Pml56 *orf2***	Forward	TGAGGATCCATGCCTTTGAATGCTCTTGCAA
**Pml56 *orf2***	Reverse	TGAGAATTCTCATTAGGGGAGATCAGATACT

1.8 Supplementary Table 3: List of all transgenic lines selected and used in the experiments, along with their corresponding abbreviations.

Transgenic plants used		
**Construction**	**Original line name**	**Corresponding name**
**pML6**	9.6.1	A1
	9.6.10	A2
	17.5.2	B1
	17.5.10	B2
	20.5.9	C1
	20.6.3	D1
	20.6.7	D2
	20.7.7	C2
**pML56 *orf2***	3.6.5	A
	3.8.4	B1
	3.8.9	B2
	11.10.3	C1
	11.10.8	C2
	11.10.10	C3

Homozygous third-generation plants were selected, and the original name was simplified to correspond to the independent line chosen for further work with each transformed plant.

1.9 **Supplementary Table 4:** Root length measurements (cm) of the different constructions without treatment (MS) and with UV treatment (UV-C) after 14 days of controlled growth.

**Root length pML6**						**Root length pML56 orf2**					
**Treatment**	**Line**	**Mean**	**Standard deviation**	**Minimum**	**Maximum**	**Treatment**	**Line**	**Mean**	**Standard deviation**	**Minimum**	**Maximum**
**MS**	A	4,905	1,516	1,335	6,090	**MS**	A	4,251	1,928	1,322	6,881
	A	6,436	0,389	6,000	6,948		B	5,683	1,150	3,153	6,706
	B	5,774	0,389	5,254	6,163		B	5,534	0,429	4,696	5,923
	B	4,593	2,004	1,000	6,888		C	4,841	1,596	1,061	7,974
	D	5,632	0,719	4,606	6,258		C	4,159	1,610	1,813	6,896
	C	5,289	1,049	3,936	6,665		C	6,081	1,151	3,917	7,865
	WT	5,469	0,931	4,000	7,027		wt	5,721	1,236	2,961	8,015
	total	5,332	1,256	1,000	7,027		total	4,924	1,639	1,061	8,015
**UV**	A	3,997	1,775	0,909	6,907	**UV**	A	4,586	0,887	3,524	6,093
	A	4,416	1,188	3,301	6,066		B	3,693	1,057	1,478	5,171
	B	3,766	1,256	1,581	5,503		B	4,074	0,873	2,952	5,804
	B	3,610	1,879	1,298	6,535		C	4,235	1,014	2,409	5,648
	D	4,467	1,572	2,488	6,548		C	4,206	0,607	2,876	5,038
	C	3,734	1,369	1,919	6,094		C	4,555	0,871	3,282	6,008
	WT	3,962	1,276	1,307	6,083		wt	3,956	1,108	1,648	6,083
	total	3,894	1,418	0,909	6,907		total	4,157	0,969	1,478	6,093
